# Atlanta Medical College

**Published:** 1857-12

**Authors:** 


### Atlanta Medical College.

The chair of Obstetrics and Diseases of Women and Children having become vacant by the resignation of J. Boring, M. D., it has been filled by the appointment of Thomas S Powell, M. D., of Sparta, Geo., a gentleman of high character and distinction in the profession, who will doubtless occupy the place with credit to himself, and to the advancement of the interests of the Institution. It will be perceived from the recent announcement of the Dean, that there continues to be in certain quarters a most singular misapprehension of the principles that govern and direct the College, in reference to its term of Lectures. *Again* and *again* we state that the Atlanta Medical College has only *one* session during the year, with the *full* interval between the Courses. The applicant for the degree must have arrived at the age of twenty-one years, and must be of good moral character; he must have been engaged in the study of medicine the usual time required by other respectable schools of Medicine, under the direction of a competent instructor; must have attended two full courses of Lectures in this Institution, or one in this and one in some other accredited school of Medicine, and in addition to these requisitions, the Faculty reserve the right in all instances to revoke the degree when sufficient evidence is afforded that he upon whom it was conferred is engaged in irregular and unprofessional practices.

Based upon principles equally high with those of any other Institution in the land, it has continued to progress, and is now in every particular taking a stand in the highest degree creditable, notwithstanding it has had to encounter a most invet erate and *unscrupulous* war from one or two *rival* schools, who happened to be a little in advance in the period of their establishment.

